# Retaining Critical Therapeutic Elements of Behavioral Interventions Translated For Delivery via the Internet: Recommendations and an Example Using Pain Coping Skills Training

**DOI:** 10.2196/jmir.3374

**Published:** 2014-12-19

**Authors:** Christine Rini, Laura S Porter, Tamara J Somers, Daphne C McKee, Francis J Keefe

**Affiliations:** ^1^University of North Carolina at Chapel HillGillings School of Global Public HealthDepartment of Health BehaviorChapel Hill, NCUnited States; ^2^UNC Lineberger Comprehensive Cancer CenterChapel Hill, NCUnited States; ^3^Pain Prevention and Treatment Research ProgramDepartment of Psychiatry & Behavioral SciencesDuke University Medical CenterDurham, NCUnited States

**Keywords:** psychotherapeutic processes, cognitive behavioral therapy, Internet, eHealth, intervention, treatment efficacy, musculoskeletal pain, osteoarthritis

## Abstract

Evidence supporting the efficacy of behavioral interventions based on principles of cognitive behavioral therapies has spurred interest in translating these interventions for delivery via the Internet. However, the benefits of this dissemination method cannot be realized unless the translated interventions are as effective as possible. We describe a challenge that must be overcome to ensure this occurs—Internet interventions must retain therapeutic components and processes underlying the success of face-to-face interventions on which they are based. These components and processes vary in the ease with which they can be translated to the online environment. Moreover, some are subtle and may be overlooked, despite being recognized as essential to the success of face-to-face interventions. We provide preliminary guidance for retaining critical therapeutic components and processes in the translation process, using Pain Coping Skills Training for osteoarthritis pain to illustrate methods. Directions for future research are also discussed.

## Introduction

Interest in developing and disseminating effective behavioral interventions is driven by the critical role behavior plays in the most prevalent physical and mental health problems in the United States and elsewhere [1,2]. Empirical support for the efficacy of behavioral interventions has been shown in research focused on disease prevention (eg, seeking to increase health promoting behaviors or to decrease health-damaging behaviors [3-6]) as well as research focused on helping people manage symptoms of diagnosed physical and mental health problems (eg, persistent pain or depression [7-10]). For example, studies of behavioral approaches to treating persistent musculoskeletal pain, cancer pain, and other types of persistent pain are yielding growing evidence for the efficacy of interventions based on cognitive behavioral therapies (CBT; eg, [9,11-13])—the most commonly researched and used psychotherapeutic approach [10]. Increasing awareness of the individual and public health risks associated with pharmacologic treatments for persistent pain [14,15] may add impetus to efforts to develop evidence-based behavioral pain treatments.

Despite the growing number of empirically supported behavioral interventions, only a small proportion of people who could benefit from them are currently able to access them. Barriers that limit access to these interventions are often interrelated and include difficulties integrating them into clinical care (eg, due to reimbursement problems or misconceptions held by health care providers about their efficacy or ideal use), the lack of available therapists in some geographic regions, the substantial time and resources needed to deliver them, the shortage of qualified therapists, and the need for people to travel to in-person sessions held at a set time [16,17].

As noted by a number of researchers [18-20], these and other barriers can be addressed by translating effective interventions for dissemination via the Internet, using interactive technologies that allow people to access them through home computers and mobile computing platforms. Internet interventions address the needs of people unable or unwilling to attend in-person treatment sessions (eg, because they are too busy, live in a remote area, lack transportation or other resources, have physical disabilities, dislike individual or group therapies, or are concerned about privacy or stigma). These individuals can complete these interventions at home, at their own pace, with access unlimited by time or day, reviewing information as often as they like. By eliminating the need for in-person meetings, Internet interventions also address barriers related to the substantial time and resources needed to deliver interventions in a face-to-face setting.

However, fully realizing these benefits requires that both interventions be efficacious. Evidence shows that Internet interventions can produce effect sizes comparable to effects of face-to-face interventions [21-25] or other forms of treatment, including medications [26]. Yet, their effects can also be disappointing even in studies that are relatively strong methodologically (eg, [27-29]). Small effect sizes suggest room for improvement, as do generally high attrition rates. One fundamental problem that may contribute to insufficient efficacy involves the extent to which Internet interventions maintain the integrity of the face-to-face interventions on which they are based. Some key components and processes of CBT-based therapies have been widely studied (eg, ways to increase self-efficacy for engaging in a behavior [30-32]). Others are less well understood (eg, subtleties in the ways that well-trained therapists interact with participants to increase engagement in intervention activities). Consequently, some of these components and processes will be more difficult than others to reproduce online. The goals of this paper are to highlight the importance of ensuring that critical therapeutic components are present and therapeutic processes are engaged when translating face-to-face interventions for Internet delivery, to provide preliminary guidance for doing so, and to describe directions for future research to support this effort.

CBT-based interventions share the assumption that people’s behavioral and emotional responses are determined not solely by objective features of the world (eg, lack of reinforcement for remaining active), but rather by how people construe them; that is, their cognitive appraisals. Therapeutic components and processes of these interventions and typical methods through which they are delivered in face-to-face interventions arise from this assumption. Accordingly, these interventions typically teach participants to use skills for identifying, reality testing, and changing maladaptive cognitions (eg, beliefs, expectations, attributions) and behaviors (eg, a sedentary lifestyle, fearful avoidance of activities they are capable of doing) that contribute to current physical or psychological symptoms or increase risk for future symptoms [33,34].

In this discussion, we will use Pain Coping Skills Training (PCST) for osteoarthritis (OA) pain [35] as a case study to illustrate methods for translating a face-to-face CBT-based intervention for delivery as an Internet-based program. PCST is most often presented by a trained therapist in an individual or group setting over the course of 10-12 weeks. The skills taught in PCST have been shown to reduce pain and disability caused by OA, cancer, and other disorders [36-41]. They target cognitions involving pain catastrophizing (exaggerated negative beliefs about pain [42]) and behaviors involving learned, maladaptive responses to pain such as spending excessive time reclining. For example, people with persistent pain engaging in PCST learn how to pace physical activities so as to gradually increase their activity and return to activities they found meaningful and enjoyable (called “activity pacing” or “activity-rest cycling” [43,44]).

## Translating Face-to-Face Cognitive Behavioral Interventions for Internet Delivery

### Overview

This section summarizes important therapeutic components and processes in CBT-based interventions, as they are applied in PCST, describes methods used to engage them in face-to-face PCST, and explains our methods for maintaining their integrity when translating PCST to an Internet intervention called PainCOACH. Rather than providing an exhaustive list of all possible methods, our intent is to offer a case study and to encourage research to guide development of best practices for future translation efforts. We begin by summarizing the structure of the PainCOACH program and our general translation approach and then discuss decisions we made about how to implement specific therapeutic components and processes of face-to-face PCST in the online environment.

### PainCOACH Structure

PainCOACH is an automated program (ie, it does not include therapist contact) that includes eight 35- to 45-minute training sessions, each teaching a cognitive or behavioral coping skill drawn from face-to-face PCST. Participants complete one session per week over 8 weeks. Session 1 starts with an overview of the PainCOACH program, PCST, and the intervention’s therapeutic rationale (a simplified version of gate control theory) [45]. This overview is followed by training in the first pain coping skill: progressive muscle relaxation. Sessions 2-7 teach, respectively, brief relaxation skills (ie, “mini-practices”), activity-rest cycling, pleasant activity scheduling, cognitive restructuring (“coping thoughts”), pleasant imagery, and problem solving. The eighth session consolidates learning and teaches strategies for long-term skill use. Between sessions, participants are asked to practice their newly learned skill and any skills they learned in past sessions.

The program also includes supplementary modules to enhance engagement and facilitate practice. One of these modules (COACHtrack) allows participants to self-monitor their progress by reviewing and changing practice goals, recording practices and “coping confidence” (self-efficacy), viewing graphic summaries of progress over time, and managing automated practice reminders. A second module (COACHchat) allows participants to read about others’ experiences using the pain coping skills and to submit descriptions of their own experiences. COACHchat was designed to provide some of the benefits of observational learning [46] that people get in face-to-face PCST when it is provided in small groups. Finally, the program includes a module (MyCOACH) that provides information about the program, the study team, and actions to take in a medical or mental health emergency.

**Figure 1:**
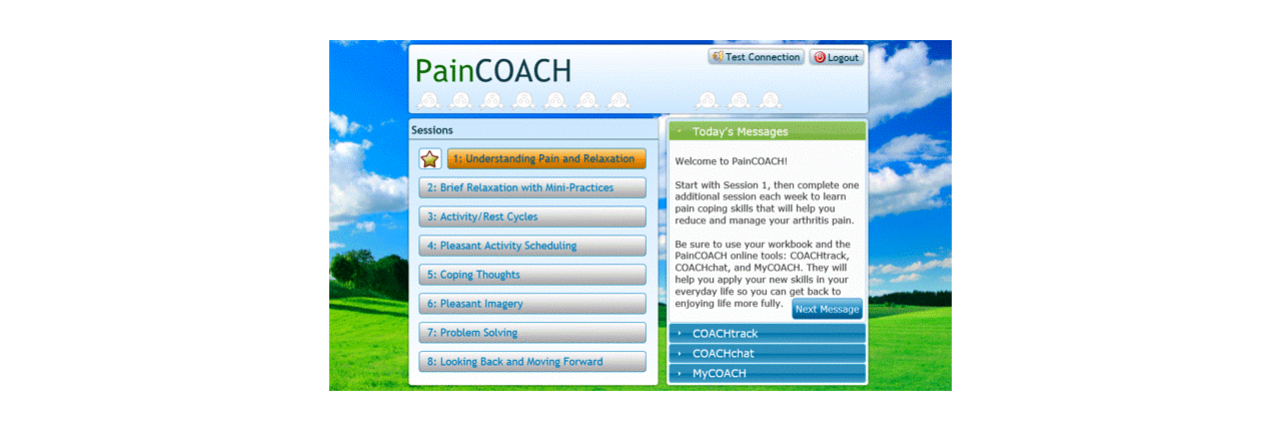
PainCOACH Home Page.

The PainCOACH home page (Figure 1) provides a portal to these modules, the training sessions, and other program features. One additional feature on the home page is a “toolbox” icon that appears next to each session once it has been completed. Selecting the toolbox gives participants easy access to segments of completed training sessions they might like to review. The home page also displays reminders and encouraging messages as well as badges participants earn by completing training sessions and selected tasks (eg, practicing skills, or reading or posting experiences in COACHchat).

The modules, sessions, and features are controlled by a programming component that applies an “expert systems” approach [47]. That is, it pairs decision rules (in the form of tailoring algorithms) with a knowledge database to simulate the behavior and judgment of an expert—in this case, a highly trained therapist experienced in delivering face-to-face PCST. The decision rules customize (tailor) participants’ experience in the program based on their responses and progress through the program. Examples of tailoring are provided in the next section.

The program was developed using standard usability guidelines [48], to ensure that it would be easy to use and appropriate for a diverse population (eg, consistency in screen design and navigation, easy-to-read text, understandable language). Usability was also enhanced by ensuring that each of PainCOACH’s eight sessions share a similar structure. Briefly, sessions begin with a review of practice completed in the prior week (except in session 1, where this segment is replaced by the introduction). Next, a new skill is introduced and participants are given the chance to try it and to evaluate their experience. After tailored feedback and remediation are provided, participants are reminded of the importance of practicing learned skills. They are taught strategies for prompting the practice of each new skill in the coming week. Sessions end with a reminder to complete the subsequent session in a week and to use COACHtrack and COACHchat.

### Translation Approach

Our challenge was to translate a highly interactive, collaborative face-to-face intervention to an automated Internet intervention, using technology to detect and address problems that would normally be handled by a trained therapist. For instance, in a face-to-face setting a therapist can monitor participants’ verbal and non-verbal cues for signs that they do not understand or agree with information being communicated—problems that would reduce the efficacy of the intervention. Our task was facilitated by the fact that we began with a well-characterized intervention. In addition, we relied on specialized clinical expertise to ensure that activities participants completed in PainCOACH were as likely as possible to have the same therapeutic effects as activities they would complete in face-to-face PCST; our development team included 4 clinical psychologists with over 90 years of combined experience delivering face-to-face PCST and similar therapies—our expert therapists. Discussion of our translation process highlights some of the critical guidance they provided.

Translation began with screen-by-screen plans laying out the PainCOACH program’s content and function, based on content from materials and scripts used in face-to-face PCST sessions. To ensure that participants would not only learn about skills, but also master how to use them, these initial plans were guided by principles of Social Cognitive Theory [46,49], adult learning [50,51], and multimedia instruction [52]. For instance, this body of literature led us to include simple interactive exercises to enhance mastery of new skills, persuasive arguments regarding participants’ ability to engage in target behaviors (social persuasion), and social modeling. With respect to the latter, we supplemented educational content with information presented by “characters”; that is, actors (an African American man, a Latina woman, and a white woman) selected to represent average members of our population in terms of their physical appearance and age. Each was given a name and persona that carried throughout the program. They reinforced main points and modeled use of skills in daily life, including barriers to using skills and ways to overcome those barriers.

The development team then met to refine the initial plans, discussing intervention components or processes that posed special challenges for translation. A primary focus of these discussions was to develop solutions that ensured the program would engage therapeutic components and processes present in face-to-face PCST, as shown in Table 1, column A. In most cases our solutions represented our expert therapists’ clinical judgment because there is currently little empirical evidence to guide these kinds of decisions. We incorporated our expert therapists’ knowledge and experience into the PainCOACH program through its content libraries and “expert systems” tailoring algorithms, described above. At times, we tried to ensure the user experience would be consistent with features of face-to-face PCST (eg, in terms of the information and feedback provided, how it was worded, training techniques, and decision rules) [47]. At other times more adaptation was needed. We describe informative examples in the next section.

### Translating Intervention Content

As shown in Table 1, column A, the first therapeutic component of PCST (and other CBT-based interventions) involves providing participants with relevant knowledge. In PCST, this knowledge begins with a plausible therapeutic rationale [53] explaining the causal link between use of pain coping skills and the ability to attain positive, desired outcomes (eg, reduced pain). The rationale provides a framework for learning skills and promotes a sense of control and positive outcome expectancies, leading to better treatment outcomes [54,55]. For PCST, the rationale uses a simplified description of Melzack and Wall’s [45] gate control theory to explain how behaviors, cognitions, and emotional responses can affect the experience of pain [53]. Additional knowledge provided in face-to-face PCST sessions focuses on explaining the skills, and providing written materials and graphics to illustrate concepts and reinforce learning.

One goal of translating this educational content to PainCOACH was to enrich it with multimedia capabilities of the online environment. Guided by usability guidelines as well as principles of adult learning [50,51] and multimedia instruction [52], animation and graphics were strategically incorporated to draw attention to key points, and simple interactive exercises were used to support learning and engagement. Participants controlled the pace of learning by using simple buttons (eg, “next” or “back”) to advance through screens that presented “chunks” of related information. In addition, research on relational agents and participant input led us to attempt to increase engagement, accountability, and adherence by minimizing onscreen text and instead having a female virtual Coach [56,57] guide participants through the PainCOACH program. An audio track of the Coach’s voice runs throughout the program. She greets participants at the beginning of each session and provides verbal instruction, feedback, and encouragement throughout the session. Her manner is conversational, empathetic, and warm. Pedagogical agents with these kinds of characteristics can promote better learning by cueing people to respond as if they are engaging in a social interaction, in turn promoting social bonding and greater motivation and cognitive processing [58-60]. All audio and onscreen text used simple, conversational language.

During the translation process, our team’s expert therapists identified the concepts or skills participants were most likely to have concerns about. Rather than having concerns linger and interfere with learning, screens addressing common concerns are used in key places throughout the program. In some places they are presented on buttons participants can press to get more information, and the responses they get are presented using language trained therapists would use in face-to-face PCST. Alternatively, in some parts of the program we addressed common concerns by having our “characters” provide social persuasion and modeling. For instance, if our team’s expert therapists indicated that some participants question the value of a particular skill, we might have a character appear onscreen talking about how he questioned its value but then tried it and found it to be helpful (eg, “I didn’t think this would work for me, but I decided to give it a try…”). Of course, it is possible that some participants will have concerns we did not anticipate and address; this is a downside of an automated program. However, because we were guided by therapists with considerable experience delivering PCST, we were confident we had covered the most common concerns that could interfere with learning.

One skill, cognitive restructuring, involves teaching participants to recognize and challenge overly negative thoughts that affect their pain. It presented a challenge to translate to PainCOACH because it is relatively abstract and therefore potentially difficult for participants to understand and apply. We addressed this challenge, in part, by breaking education about this skill into two sessions. First we introduced the concept of “unhelpful automatic thoughts” with animated examples and social modeling to show examples in the context of daily life. Then participants were asked to use a worksheet (provided in a printed program workbook) to track their unhelpful automatic thoughts, the situations in which they occurred, and any emotional and physical consequences they noticed in the coming week. We reasoned that completing this homework and receiving feedback on these initial experiences would set the stage for the next session’s training in how to reduce unhelpful automatic thoughts using “coping thoughts” (ie, cognitive restructuring), which included interactive exercises that helped participants apply this new educational material to experiences they had tracked in their prior week’s homework.

It is important to note that education alone is not sufficient for ensuring mastery of pain coping skills, nor is it sufficient for ensuring that participants can use the skills appropriately and consistently in daily life. Rather, education is part of a more comprehensive training approach. One distinctive feature of PCST, as with CBT-based interventions in general, is that face-to-face training is delivered in sessions that are highly collaborative (the second therapeutic component in Table 1, column A). Therapists use a variety of methods to help participants master new skills and to increase their motivation to use them regularly in daily life—a necessity if the skills are to have a positive influence on health and well-being.

Some methods therapists use to achieve these goals in face-to-face PCST are summarized in Table 1, column B, and examples of ways this component was implemented in PainCOACH are summarized in column C. For instance, therapists use behavioral rehearsal to help participants develop a first-hand understanding of how it feels to use a skill effectively. They typically model a new skill, guide participants as they complete experiential exercises (eg, role play, guided discovery, behavioral experiments), observe participants’ performance to ensure adequate learning, and provide corrective feedback and encouragement. These exercises help participants build proficiency and self-efficacy for using the skills [49] and reinforce positive outcome expectancies, in turn increasing the probability they will have positive experiences that increase their motivation to use the skills.

One example of how collaborative skills training was translated to PainCOACH is a process we referred to as “unpacking” a participant’s experience with using a new skill. Unpacking is a process through which participants learn to be aware of their emotional, cognitive, and behavioral responses to practicing a skill—how and where they are doing it, how it makes them feel, which aspects of the practice worked well or poorly, and how they might alter their practice to be more effective in the future. Its goal is to help them understand and benefit from practice. In a face-to-face PCST, the therapist would ask a series of questions to highlight features of the practice experience (eg, “what changes did you notice in your body or in what you were feeling or thinking while you practiced?”), monitoring participants’ verbal and nonverbal responses to address unspoken thoughts, concerns, or questions. These activities help participants recognize and appreciate positive experiences during practice. They also build participants’ self-efficacy for recognizing and solving problems that lead to negative experiences that can reduce motivation to use skills. In sum, unpacking is complex and subtle yet critical for learning and, ultimately, behavior change. We believe this important process is likely to be overlooked in unguided Internet interventions and that it is likely to be used insufficiently in guided Internet interventions that include minimal therapist contact [61], or when the therapist role is enacted by individuals who are not trained therapists.

For PainCOACH, we developed an interactive unpacking exercise that participants completed after practicing selected skills. For instance, participants were educated about progressive muscle relaxation in Session 1, then followed the Coach’s audio instructions to try the skill. After they finished the practice, the Coach walked them through the unpacking exercise. The first step in the unpacking exercise was designed to address a challenge that the team’s expert therapists alerted us to—participants do not always notice or pay attention to sensations and other experiences they have when they first practice a new skill. This kind of self-examination is new and unfamiliar to most participants. Consequently, we chose to have our three characters model this process. Specifically, the Coach introduced the idea that people have different experiences when they try progressive muscle relaxation and then explained to participants that they could hear the characters describe their first experience with progressive muscle relaxation by selecting each character’s picture. The characters’ responses were designed to model how a person would notice and describe common positive or negative experiences with progressive muscle relaxation: William mentioned a common physical sensation (legs feeling heavy, like they were sinking into his chair), Linda noted a sense of calm that lasted even after the practice ended, and Patricia discussed being distracted by her thoughts before realizing she could put her worries aside for a while to focus on relaxing (modeling a solution).

The next step in the unpacking exercise was to allow participants to report their own experiences and receive feedback from the Coach. Continuing the session without having the Coach ask about and respond appropriately to participants’ experiences would frustrate those who had negative experiences (eg, because the Coach did not understand their problems and demonstrate concern about their learning) while missing an important opportunity to reinforce positive experiences. A responsive “dialogue” between the Coach and participants was important for building the working alliance so critical to CBT-based therapies. In order to implement this dialogue, PainCOACH’s expert system needed information that could be used to tailor the Coach’s response. We chose a simple interactive exercise to get this information. The Coach asked participants what they had experienced during their practice, and they responded by dragging and dropping boxes labeled “more of this”, “less of this”, and “no change in this” into boxes labeled with positive and negative experiences commonly reported by participants in face-to-face PCST. Thus, a participant might report a relatively positive experience by reporting increases in feeling relaxed, happy, and clear-headed and decreases in pain, stiffness, and muscle tension, with no change in other phenomena. Another participant might report a relatively negative experience, such as increases in feeling fidgety and sleepy, with no decrease in muscle tension. Participants’ responses were used by the tailoring algorithm to provide appropriate feedback (eg, reinforcing positive experiences or normalizing and addressing negative experiences). Finally, the unpacking exercise ended with the Coach introducing a list of common concerns and problems that participants could select to get reassurance, normalization, and tips on how to resolve them. The goal was to resolve that could reduce participants’ motivation or self-efficacy for using the skill. All of these steps in the PainCOACH unpacking exercise were evaluated in user testing, as described below, to ensure they were intuitive to use and made participants feel understood and supported.

The third therapeutic component of PCST (shown in Table 1, Column A) involves self-monitoring. Participants are taught to recognize pain-relevant patterns of behavior, emotions, and cognitions in their daily life. In face-to-face PCST, therapists provide worksheets to facilitate self-monitoring and discussion, in turn helping participants recognize when and how to use pain coping skills (see column B). As summarized in column C, we implemented self-monitoring in PainCOACH by having the Coach teach participants to complete worksheets (provided in a printed program workbook), illustrating this task with animated graphics, then having participants complete interactive exercises to learn how to recognize their own maladaptive behaviors, emotions, and cognitions and the situations in which they occur.

The next therapeutic component of PCST (the fourth component in Table 1, Column A) helps ensure that participants are able to use skills appropriately and stay motivated to do so over time. This is a critical part of training because health benefits require that the skills are used in daily life. Examples of ways therapists deliver this component in face-to-face PCST are summarized in column A. For instance, participants are expected to practice using the skills outside of sessions to ensure they gain experience using skills in various life situations, develop confidence in their ability to use them, and develop an ability to apply them in a flexible manner to meet varying challenges of daily life. In PCST, like other CBT-based pain interventions, participants are not expected to use all of the skills they learn all of the time. Rather, they learn to identify the skills they find most helpful and to incorporate them into a plan to prevent or manage pain flares.

It is also important to understand participants’ experiences with practice; they are not likely to keep using skills if they do not find them to be useful or if they dislike using them for other reasons. Accordingly, after participants learn a new skill in face-to-face PCST, they work with their therapist to develop goals and plans for practice. Then, in their next session, therapists and participants review whether practice sessions and goals were completed and discuss successes and/or problems participants experienced. The therapist helps participants recognize successful experiences, reinforcing them and problem solving as necessary.

Features of the PainCOACH program summarized in Table 1, column B, were designed to correspond with these important activities. The Coach explains the importance of practice early in the program. That message is reiterated throughout the program, and various program features support and reward practice. For instance, at the end of each training session the Coach suggests strategies for remembering to practice, then leads participants through an interactive exercise to set practice goals for the newly learned skill. Participants are periodically reminded to update their goals in COACHtrack, which also allows them to record practices they complete between sessions and to generate a graph showing their progress over time. They are rewarded for completing practices with badges recognizing their accomplishments. In addition, at the beginning of each session, the Coach reviews participants’ completion of practices in the prior week. She uses any practices participants entered in COACHtrack as a starting point (eg, “I see from COACHtrack that you practiced your activity/rest cycle last week…”) or allows them to indicate a number before continuing. For participants who have not practiced, the Coach provides information to troubleshoot reasons this may have occurred and reiterates the importance of practice, guided by tailoring algorithms to ensure the information and suggestions are appropriate. The wording of these communications was carefully crafted to reflect approaches therapists use in face-to-face training (eg, to develop a strong working alliance, as described next). PainCOACH also lets participants set up automated practice reminders delivered as an email, text message, or telephone message.

Session 8 of the program also promotes ongoing use of the skills through its focus on preparing participants to continue using skills over time. Participants complete exercises that involve thinking about which skills they have used most and found to be most helpful. They also play a game that asks them to consider the kinds of situations that might lead them to use different skills. It presents scenarios, asks them to select which skills they would use in the scenarios, and provides feedback on their selection of skills. Other exercises help them think about setbacks that might reduce their use of skills and how to overcome them.

The last key component of CBT-based interventions shown in Table 1 [45,50,62], column A, is the working alliance that therapists develop with participants. Specifically (as summarized in column B), they provide treatment in a warm, accepting manner that communicates partnership in the process of behavior change. They assess and monitor participants’ ongoing level of understanding, motivation, and investment in the intervention and the factors that interfere with treatment success. They also adjust the intervention as needed, based on their knowledge of psychosocial factors that affect participants’ engagement in treatment. Taken together, these methods enable the development of a working alliance.

It is currently unclear whether it is critical to develop this kind of alliance in Internet interventions. Some research suggests that it might not be [63,64], but other research has found that developing a greater alliance is associated with better outcomes in Internet interventions [65]. More research is needed to clarify whether the importance and nature of the working alliance differs in face-to-face versus Internet interventions. When translating face-to-face PSCT for delivery in PainCOACH, we considered it to be important and took steps to ensure participants developed the sense that behind the program text, audio, and exercises was an empathetic therapist who understood them, cared about their pain, and was collaborating with them to find ways to manage it (summarized in column C). For instance, our decision to have the program led by a virtual Coach was motivated in large part by a desire to facilitate development of a strong working alliance. As noted above, this program feature was also expected to increase engagement, accountability, and adherence [53,54]. Images of the Coach (which change so that her body language and facial features correspond to ideas being expressed) appear onscreen frequently. Her manner is conversational, empathetic, and warm. She greets participants when they begin sessions and speaks directly to them throughout the program, including setting up an appointment with them to return for the next week’s session. Tailoring algorithms are used to ensure her feedback and responses are personalized so that participants feel that they are understood. Her dialogue was carefully crafted to use techniques that therapists are trained to use to promote development of a working alliance in face-to-face PCST (and other CBT-based interventions).

**Table 1:** Therapeutic components and processes of cognitive behavioral therapies.

A	B	C
Therapeutic components of CBT interventions	Methods for delivering them in face-to-face interventions	Examples of methods for delivering them in Internet-based interventions
1. Relevant knowledge (education) Therapeutic rationale: Simplified explanation of Gate Control Theory [45] and how cognitions, behaviors, and emotions and description of how changing them would achieve treatment goals. Information about skill: Description of skill, referencing processes described in rationale, along with explanation of how and when it is used in daily life.	Therapist provides verbal education in highly interactive and collaborative manner	Multimedia education; Interactive exercises to assess learning and provide remediation
		Concerns addressed through text covering “frequently asked questions”, social modeling (ie, stories told by characters similar to the target audience), or providing ability to ask questions and get personalized response (eg, via email)
		Ability to revisit material as needed
	Therapist provides written educational materials to reinforce learning	Workbook or ability to print computer-tailored materials
	Graphics used to illustrate concepts and reinforce learning	Graphics enhanced with audio, animation, and testimonials (to provide social modeling)
2. Collaborative skills training, focused on developing mastery of skills and self-efficacy for using them in daily life	Therapist models skill to demonstrate its proper use	Skill modeled in video or with graphics showing therapist or simulated participant (for social modeling)
		Ability to revisit material as needed
		Easy to access supportive resources (eg, audio recordings to guide practices available for download or accessible through mobile technologies)
	Therapist guides participant through coping skills practice (behavioral rehearsal)	Program guides participant through behavioral rehearsal
	Therapist observes performance to ensure adequate learning, providing corrective feedback	Interactive exercises help participant identify problems experienced during behavioral rehearsal
		Computer-tailored corrective feedback
	Therapist solicits information about participant’s experience with behavioral rehearsal	Interactive exercises to gather information about participant’s experience with behavioral rehearsal
	Therapist highlights positive experiences	Interactive exercises to help participant recognize positive experiences
	Therapist provides feedback about performance to promote self-efficacy and positive outcome expectancies	Computer-tailored feedback appropriate to participant’s report of problems and positive experiences during behavioral rehearsal
		Interactive exercise to assess and promote self-efficacy and positive outcome expectancies
	Therapist instructs participant in how to use skills and associated worksheets outside of sessions	Multi-media instruction in how to use skills and worksheets outside of sessions
		Examples of skill and worksheet use modeled by simulated participants
		Interactive exercises to help participant develop personalized plan describing where and when skills and worksheets will be used
	Participant practices skill outside of session	Participant sets up and receives automated practice reminders
		Participant practices skill outside of session
	Participant tracks practices and experiences with paper worksheet	Participant tracks practices and experiences with electronic application (eg, on mobile device)
		Immediate computer-tailored feedback to reinforce practice
		Graphics show progress over time
	At subsequent session, therapist reviews worksheet with participant to provide feedback regarding frequency and adequacy of skill use	At subsequent session, interactive exercises elicit number of practices completed and experiences during practices
		Interactive exercises identify and troubleshoot problems and draw attention to positive experiences
	Therapist provides feedback to reward skill practice and to reinforce future practice	Immediate computer-tailored feedback about frequency and adequacy of skill use
		Badges, unlocked features, or other rewards to recognize skill practice and to reinforce future practice
3. Self-monitoring: Recognizing and recording experiences to promote use of skills	Participant and therapist identify participant’s maladaptive cognitions, behaviors, and emotions and the internal and external cues associated with them (eg, emotions, environmental factors, social/interpersonal factors).	Interactive exercises help participant identify maladaptive cognitions, behaviors, and emotions and the internal and external cues associated with them (eg, emotions, environmental factors, social/interpersonal factors).
	Therapist shows participants how to complete worksheets that facilitate self-monitoring (eg, “thought records” used to record thoughts associated with negative responses)	Multimedia instruction in how to complete worksheets
		Electronic worksheets (eg, using mobile technologies)
4. Ensure appropriate, enduring use of skills in daily life	Therapist works with participant to set appropriate practice goals	Multimedia education on setting appropriate practice goals
		Interactive exercises help participant set goals
	Therapist works with participant to develop personalized strategies for meeting goals	Multimedia education showing strategies for meeting goals (including modeling by simulated participants)
		Ability to set up automated reminders (eg, phone calls, email, text messages, coordination with wearable monitors)
	Therapist provides worksheet to record goals and track goal-related behaviors	Participant records goal-related behaviors in program (eg, on computer or mobile device)
		Graphical display show goal attainment over time
	Therapist works with participant to identify barriers to goal attainment and problem solve as necessary	Interactive exercises to identify barriers to goal attainment, with computer-tailored guidance for how to overcome them
	Therapist works with participant to increase goals over time, being responsive to participant preferences	Participant periodically reminded to update goals
		Program assists with setting new goals using computer-tailoring based on practice history and goal attainment
	Therapist recognizes goal attainment to enhance participant self-efficacy	Badges, unlocked features, or other rewards to recognize goal attainment
	Discussion with therapist regarding possible future setbacks that could affect ongoing use of skills	Multimedia education describing possible future set-backs, modeled by simulated participants
		Interactive exercise to generate personalized list of possible future setbacks that could affect ongoing use of skills
	Therapist guides participant in developing a plan to cope with setbacks	Interactive exercise to develop plan to cope with setbacks (eg, applying research on implementation intentions to create a specific if-then plan) [62]
5. Therapeutic/ working alliance	Therapist behaviors (warmth, acceptance, collaborative approach) promote therapist-participant bond and ability to agree on goals and tasks	Use of “virtual coach” (a relational agent; [57]) to build social-emotional relationships with participant by emulating face-to-face interaction with therapist
	Therapist assesses participant’s engagement and agreement with goals and methods at various points during therapy	Algorithms evaluating participant engagement through program use, practice, and goal attainment
		Assessments to measure participant’s agreement with goals and methods at various points during therapy
		Computer-tailored messages to provide personalized feedback in a way that communicates warmth, acceptance, and collaborative approach

### User and Pilot Testing

Our iterative development process included both user testing and pilot testing, with the overarching goal of ensuring the PainCOACH program was technologically sound, engaging, and easy to understand and use by our target population—older adults with OA of the hip or knee and OA-associated persistent pain, some of whom would be rural and of low socioeconomic status.

Two rounds of user testing were conducted early in the development process with 49 participants drawn from our target population, selected to be diverse in terms of gender (female: 55%, 27/49), race (Non-Hispanic white: 51%, 25/49; African American: 49%, 24/49), education (from less than high school to graduate degrees), and experience using computers and the Internet (from none to daily). They ranged in age from 53-87 years old (with an average age of 67). Groups of participants were provided with an introduction to the PainCOACH program and study goals, then met one-on-one with a trained study staff member who observed them as they worked through selected sessions and used a semistructured interview to gather feedback. In the first round, participants provided feedback on the content and functionality of a working prototype of the program’s home screen and session 2. In the second round, participants provided feedback on the content and functionality of the revised session 2, plus two additional sessions. Across both rounds of user testing, positive feedback indicated a high degree of enthusiasm for learning PCST and using an Internet intervention for doing so. Many features of the program were well liked, including the overall look of the program, the Coach, learning about others’ experiences using PCST (ie, the characters), the information that was provided, and the skills being taught. Feedback also revealed a need for various refinements, including redesigning the home screen to clarify its features and functions, eliminating most exercises that required typing (because of confusion about using the tablet computer’s onscreen keyboard), reducing text and making it larger, and making some navigation features and exercises more intuitive.

When a working prototype of the entire program was complete, eight participants pilot tested it at home. All had OA of the hip or knee and were screened for having significant OA pain. The sample was 75% female (6/8), 63% Non-Hispanic white (5/8), and 38% African American (3/8), aged 54-76 (average age 68 years), and diverse in terms of annual income (less than US $15,000 to approximately $75,000), education (less than high school to a graduate degree), and experience using computers and the Internet (none to daily). After an in-person orientation meeting, participants were asked to complete the PainCOACH program within 2 weeks. Our goal was not to have them learn and use the pain coping skills, but rather to provide feedback on technical problems and any aspects of the program that were difficult, confusing, or frustrating to use. Pilot test participants were given a notebook with screenshots to aid in noting and reporting problems, which were communicated to us throughout pilot testing in brief phone interviews. At the conclusion of the pilot test, participants completed a questionnaire to report usability. Overall, feedback was highly positive. For instance, most participants strongly agreed or agreed that it was easy to log into PainCOACH and navigate through it, they felt confident using it, and they thought it was easy to use. All agreed or strongly agreed that the information was useful, that it made them feel that pain coping skills were important to learn, and that the exercises and stories about other people’s experiences helped them understand the information. The pilot test guided final refinements to the program, with most changes involving needing to optimize the speed at which the program’s audio downloaded and to ensure participants had a strong enough Internet connection to access the program. Once these final refinements were made, we began a small-scale randomized controlled trial to evaluate its effects on key participant outcomes, acceptability, and feasibility. Feedback from the trial, in addition to evidence for the program’s efficacy, will be used to make additional refinements for a larger scale evaluation.

## Discussion

The benefits of delivering proven behavioral interventions via the Internet are compelling and address goals related to individual and public health. However, achieving these goals requires that the Internet versions of these interventions be as effective as possible. In the present paper, we highlighted one critical, but underappreciated challenge that must be overcome to ensure this occurs—behavioral Internet interventions must retain therapeutic components and processes underlying the benefits of the face-to-face interventions on which they are based (eg, knowledge, collaborative skills training, self-monitoring, ensuring appropriate and enduring use of skills, and working alliance). In the case of CBT-based interventions, these components and processes vary in the ease with which they can be translated to an interactive, online environment. Moreover, some are subtle and may be overlooked in the translation process. Failing to engage them is likely to limit the efficacy of otherwise promising interventions, including those that are automated and therefore do not include therapist contact.

Unfortunately, little empirical evidence exists to guide decisions that must be made during the translation process. In light of the dearth of evidence, we argue that these interventions are more likely to be maximally effective if they are developed in close collaboration with expert therapists. Their professional training in the clinical application of the science of behavior change, paired with their extensive hands-on experience delivering an intervention in person, allows a development team to anticipate parts of the intervention during which participants will have problems learning and applying skills; the specific problems they will have; the best time-tested solutions for resolving the problems; and how to deliver those solutions so that participants are most likely to accept and benefit from them. Expert therapists are uniquely able to provide this critical input.

Close collaboration with expert therapists is likely to be especially critical for automated interventions that do not include any interaction with a therapist because these interventions depend solely on the adequacy of the computer program for their benefits. That is, their involvement facilitates use of an “expert systems” approach [47], which we implemented by approximating a real clinical encounter (eg, how a therapist assesses participants’ needs, makes decisions based on those needs, and provides feedback in face-to-face PCST). We have highlighted one approach, but others certainly exist and more will arise as new technologies emerge and mature. Of course, each approach must be evaluated with research. It seems likely that research could find other effective strategies for engaging therapeutic processes online. Theoretical models of behavior change developed specifically for Internet interventions may be useful as a starting point for this work [66,67], as can research showing the most effective components of behavioral interventions [68].

Indeed, it has been noted that simulating interactions as they occur in a face-to-face setting may not always be the ideal goal. Rosser et al [69] propose that differences in how people interact with other people compared to how they interact with machines—or with other people via machines—will sometimes require therapeutic methods to be adapted rather than directly translated for Internet delivery. We agree and add that methods of engaging therapeutic processes may also be fundamentally changed—and potentially strengthened—by technological capabilities of the Internet. Consider, for instance, the goal of educating participants. Compared to written materials and graphics used in the face-to-face setting, it may be possible to enhance learning and mastery of new information through use of multimedia skills training paired with experiential exercises in which participants apply new knowledge to solve problems [52,70]. Likewise, new sensor technologies and smartphone applications may enable more effective self-monitoring than paper-and-pencil worksheets. Sophisticated use of emerging technologies such as wearable sensors, natural language processing, and machine learning could make self-monitoring less burdensome and more likely to help people detect and change maladaptive behaviors. Best practices for these and other uses of technology will need to be established with research.

If PainCOACH succeeds in improving pain and related outcomes, its success will provide only indirect evidence for the importance of ensuring that CBT-based Internet interventions (and particularly automated interventions) maintain the integrity of their corresponding face-to-face interventions. The trial testing PainCOACH was not specifically designed to evaluate the program’s engagement of the same therapeutic processes as those engaged by face-to-face PCST. Conversely, if PainCOACH does not succeed in improving participant outcomes, it will not necessarily invalidate our argument; there are many reasons an Internet intervention may fail. Thus, the strongest foundation for development of CBT-based Internet interventions requires research focused specifically on evaluating the importance of each therapeutic component and establishing best practices for translating them to the Internet. Similar to usability standards that help researchers develop Internet-based interventions that are easy to use and engaging, it should be possible to develop empirically supported standards for translating or adapting critical therapeutic components and processes of face-to-face CBT and other therapies to the Internet. Innovation will always be important, but these kinds of standards would be an invaluable starting point to ensuring more efficient use of resources.

## Abbreviations

CBT: cognitive behavioral therapy
OA: osteoarthritis
PCST: Pain Coping Skills Training
